# Trichoscopy-Derived Hairline Recession Equivalent in Monitoring Frontal Fibrosing Alopecia

**DOI:** 10.1159/000524127

**Published:** 2022-05-19

**Authors:** Justyna Sicińska, Michał Kasprzak, Irena Walecka

**Affiliations:** ^a^Department of Dermatology, CSK MSWiA/CMKP, Warsaw, Poland; ^b^TrichoLAB, Warsaw, Poland; ^c^Dermiq Medical Center, Warsaw, Poland

**Keywords:** Frontal fibrosing alopecia, Trichoscopy, Follicular map, Hair-to-hair matching, Hairline recession equivalent

## Abstract

**Introduction:**

Frontal fibrosing alopecia (FFA) is a relatively recently described scarring hair loss condition. Frontal hair recession is observed in a vast majority of patients; other scalp areas may be included. Assessment of hair loss progression in FFA remains challenging mainly due to difficulties in unambiguous determination of the hairline. Various patterns of scarring and subtle progression rate are among factors which make naked-eye observations of limited use.

**Methods:**

Trichoscopy of the frontal hairline with hair-to-hair matching was conducted in patients with FFA patients with disease progression and clinically stable hairline. Hair loss was assessed based on analysis of trichoscopy-derived follicular maps. A relative hair density loss was calculated, and the hairline recession equivalent (HRE) was proposed as a novel hair loss progression measure.

**Results:**

Two patterns of hair loss were observed: one with significant decrease of hair density within a width of 1 mm and one with diffuse loss within a width of 10 mm.

**Conclusion:**

The hair density profile may be a useful tool to characterize different disease progression patterns. The HRE is potentially a very accurate and sensitive parameter to quantify local hair loss progression in FFA.

## Introduction

In the last decade, a significant increase in the incidence of frontal fibrosing alopecia (FFA) cases has been observed [1, 2, 3, 4, 5, 6]. Currently, there is no treatment consensus for FFA, but certain therapies have been reported as beneficial [7, 8, 9, 10]. Scarring in FFA causes the anterior hairline to recede [11, 12, 13, 14]. The same process is responsible for the appearance of patches in less frequent patterns of FFA, i.e., pseudo-fringe pattern and cockade-like pattern [15, 16, 17]. Precise assessment of hair loss progression in FFA has remained a challenge for years. Holmes et al. [18] proposed the Frontal Fibrosing Alopecia Severity Index (FFASI), while Saceda-Corralo et al. [19] proposed the Frontal Fibrosing Alopecia Severity Score (FFASS). Both indexes are multifactorial with hairline recession included as one of the parameters.

In most cases, difficulty in assessing this parameter arises from a lack of specific anthropometric reference points on the forehead and a poorly defined hairline. Measurement of hairline shift alone may not be an efficient method to assess hair loss progression in a percentage of FFA patients. In this study, hairline analysis comprised trichoscopic examination with hair density assessment. The objective of the study was to observe modification of patient's follicular map as an expression of the disease activity.

Over the last two decades, trichoscopy has become a standard procedure for clinicians who treat FFA patients. Trichoscopic findings such as loss of this and vellus hairs, perifollicular erythema, and perifollicular scaling over the band-like alopecia area enable a bedside diagnosis [20, 21, 22]. Further loss of hair follicles and subsequent scarring prove progression of the disease. Detailed assessment of this phenomenon provides information on the disease course.

Investigators will welcome the development of a globally relevant, validated method which reflects the key morphologic feature of FFA, namely hair loss [23]. The ideal tool for monitoring FFA should be efficient and simple, yet precise and effective in assessing therapeutic response, especially in clinical studies [24, 25].

## Methods

Six patients were selected among FFA outpatients of the CSK MSWiA/CMKP clinic. The inclusion criteria were clinically evident and pathologically confirmed FFA, no ongoing or new hair treatments, no clinically noticeable ongoing hairline regression in the past 12 months (clinical stability was defined based on the previous visit 1 year earlier). The exclusion criteria were neurological disorders with tremors making precise imaging impossible, extremely low density within the hairline, predominance of gray hair. All patients were under routine yearly trichoscopy monitoring. The hereafter presented results are based on two consecutive trichoscopy examinations performed at a 12-month interval on each of the patients. All examinations took place between October 2019 and January 2021.

USB microscope with imaging software (FotoFinder leviacam® and TrichoLAB Suite with Virtual Tattoo technology) was used to register trichoscopy images of the frontal hairline with hair parted along the midline. The frontal hairline is always affected in this disease, and because of this fact, the forehead is the main point of interest for our study.

In each patient, the examination spot was marked with four ink dots spaced every 1 cm along the midline, with the lowest one below the hairline. The dots were used for camera positioning when taking three partially (30%) overlapping trichoscopy images sized 0.6 cm × 1.8 cm. The images combined together covered an area of 2.4 cm^2^ (0.6 cm × 4 cm, with the long axis along the midline). This imaging was repeated 2 more times after hair re-parting to ensure good visibility of all hair. During the follow-up examination after 12 months, clinical naked-eye assessment was conducted. Additionally, imaging of exactly the same spot was achieved with a two-step procedure. First, the location was obtained by placing ink dots at approximately the same distance from the nasion as during baseline examination. Then, the images were micro-aligned to match the follicular map pattern of the baseline images using TrichoLAB Virtual Tattoo. Once aligned, the same imaging setup and procedure were used as for the baseline examination. The TrichoLAB system was used to process all the registered images and to perform the statistical analysis.

The steps described above may be reproduced with any videodermoscopic equipment (e.g., Handyscope Heine) capable of skin imaging with magnification suitable for hair detection (20–50×). Finding the same imaging spot without Virtual Tattoo technology may be achieved by reference to pigmented nevi, anthropometric points, or by micro-tattooing. Once the baseline and the follow-up images of the same hairline test spot are done, the position of hair follicles can be identified manually.

For the qualitative evaluation of hairline change, the baseline and the follow-up images were aligned so that their follicular maps matched perfectly. The comparative hair-to-hair (H2H) matching analysis, based on the follicular map concept, allowed visual identification of all preserved, missing, and new hair shafts [26]. The quantitative comparison of baseline and follow-up examinations was performed using hair density profiles − charts of local hair density as a function of the distance from the nasion. To assess the overall rate of hair loss in the hairline area, the lost hair count was normalized to the average number of hair shafts per millimeter in the frontal scalp at the baseline.

The resulting measurement (expressed in millimeters) is further referred to as hairline recession equivalent (HRE). In an oversimplified model of FFA affecting only the lowest located shafts, HRE is equal to the actual hairline shift expressed in millimeters.

Error propagation analysis shows that HRE uncertainty due to hair misdetection is below 0.1 mm as the terminal hair detection efficiency in matched analysis is above 99% [27]. The H2H matching concept eliminates influence of typical systematic effects like incorrect alignment of follow-up versus baseline examinations or scalp skin stretching. The main factor limiting the precision of HRE is therefore the fluctuation of the number of hairs that have fallen out in the studied part of the hairline section between the baseline and the follow-up examinations. In accordance with the Poisson distribution, the standard deviation was estimated at the level of 0.4–0.5 mm in the presented examples.

## Results

The study was performed on six females with FFA (mean age 58 years, range 49–68) with a prevailing diffuse pattern within the observed area. After 12 months, no patient presented further progression upon clinical naked-eye examination.

**Fig. 1:**
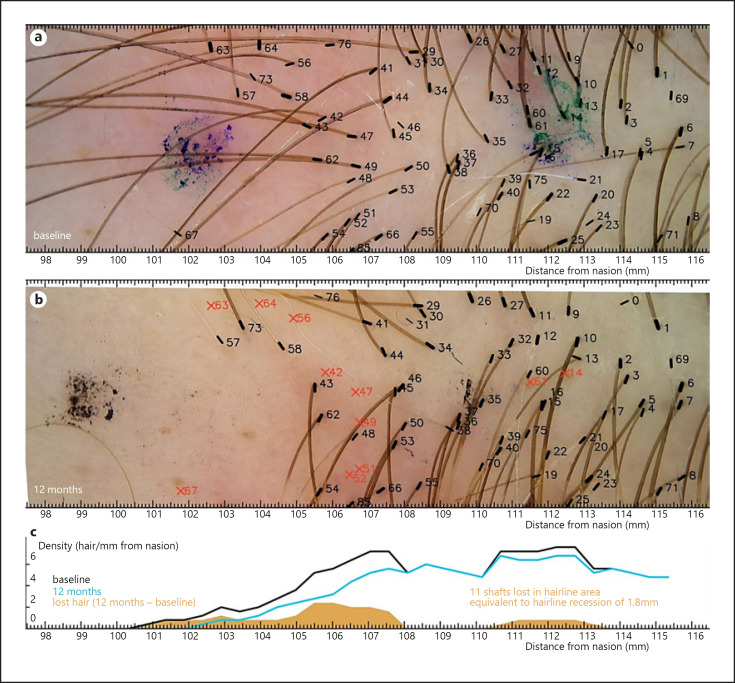
**a–c**Trichoscopic hairline monitoring in FFA patient A with narrow-strip hair loss. The baseline image aligned with the follow-up image and hair density profile. Loss of 11 hair shafts (positions of lost shafts marked with red x) with no new hair in the field of view. The majority (nine out of 11) of lost hair shafts were located in a narrow (circa 1-mm-wide) strip behind the hairline. The majority of shafts forming the hairline were preserved. Eleven lost hair shafts relative to the average of 6.1 hairs/mm gives an HRE of 1.8 ± 0.5 mm. The marker dots were used for approximate alignment only.

**Fig. 2:**
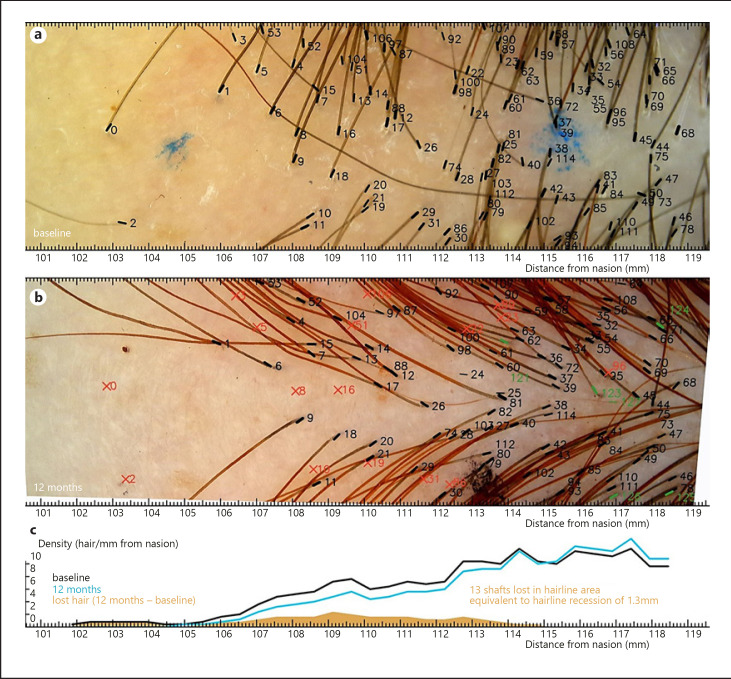
**a–c**Trichoscopic hairline monitoring in FFA patient B with wide-strip hair loss. The baseline image aligned with the follow-up image and hair density profile. Loss of 16 hair shafts (positions of lost shafts marked with red x) slightly compensated by appearance of new hair, mainly further from the hairline (marked green). The hair loss occurred within a 1-cm wide-strip behind the hairline. Loss of the lowest located, hairline-forming shafts gives the impression of a hairline shift of circa 4 mm. The net loss of 13 hair shafts relative to the average of 10.2 hairs/mm gives an HRE of 1.3 ± 0.4 mm. The marker dots were used for approximate alignment only.

Nonetheless, H2H image analysis revealed significant hair loss in 4 patients within the imaged area as presented in Figures 1 and 2. Figures show only the lowest located, hairline-centered images (out of 3) as the other images showed no signs of FFA activity. The other collected images of the remaining 2 patients in this study do not provide additional information.

**Fig. 3:**
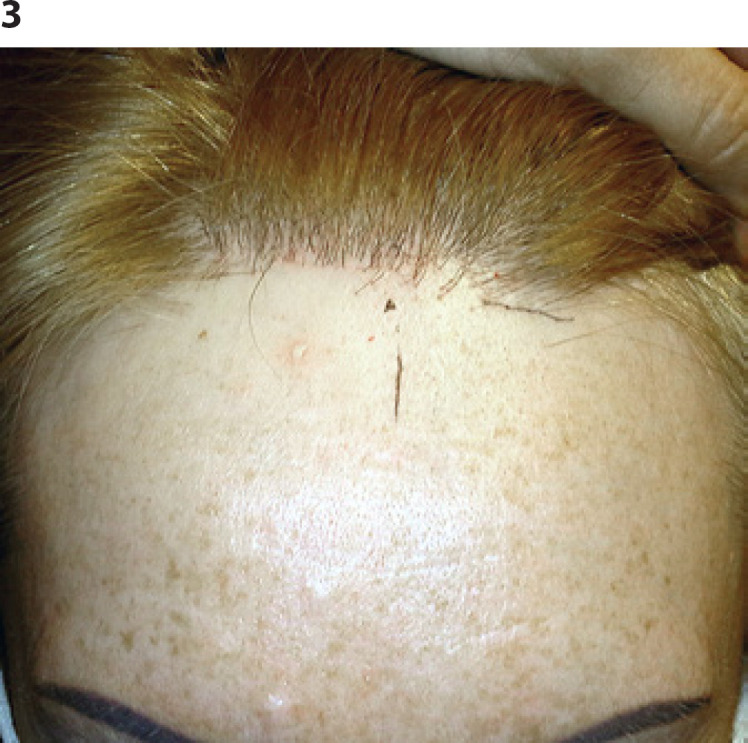
Clinical picture of patient A.

**Fig. 4:**
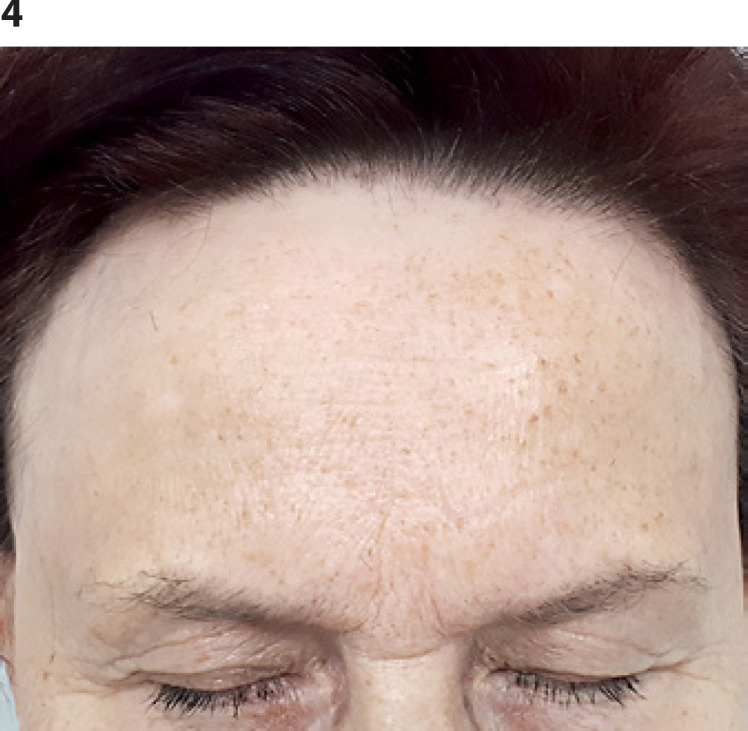
Clinical picture of patient B.

In two of these cases, the hair loss occurred within a narrow strip upward from the hairline (Fig. 1). In the other 2 cases, the hair loss of single shafts occurred in a diffuse manner (Fig. 2). Clinical pictures of the 2 patients are presented in Figures 3 and 4. The charts in both figures present comparisons of baseline and follow-up hair density profiles.

## Discussion

The evolution of hairline in FFA patients was studied with trichoscopy over a period of 12 months. The H2H matching and aligned follicular maps showed significant hair loss in patients with no observed clinical progression. The variety of hair loss patterns made it difficult to quantify the phenomenon clinically and to conclude if there was a hairline shift.

The quantification issue was addressed with the use of hair density profiles, presenting the density of hair across the hairline. Short and high curves in the profile chart were obtained in patients with hair loss affecting the lowest located frontal hair shafts (Fig. 1), while long and flat curves corresponded to “wide-strip” diffuse hair loss (Fig. 2). As only a small group of FFA patients has been observed, a variety of other patterns might appear in the future as more patients are studied.

To address the question of hairline recession, the authors propose a new parameter: HRE. This parameter is the number of lost hair shafts normalized to the average density of the frontal scalp. Although basic stability and reliability checks of HRE have been performed, further extensive study is required to validate it across bigger FFA patient groups, in other hair loss conditions as well as in healthy individuals.

Observation of the hairline in combination with density analysis provides deeper insights into disease progression. This approach seems especially appropriate in patients with diffuse variant and in patients with pseudo-fringe pattern.

Figure 2, in addition to missing hair, shows some new hair shafts that emerged in follow-up examination and were absent in the baseline. This led to the follow-up hairline profile curve (blue) running above the baseline curve further up the frontal scalp. Some level of hair fluctuation is normal due to hair cycling and does not affect the HRE measurement significantly, but large fluctuation due to, e.g., telogen effluvium could reduce HRE measurement precision. Further study is required to assess the HRE behavior for TL and other conditions.

The challenges for the method include the need for professional equipment and the spot character of control examinations if only one spot is assessed. An investigator should also bear in mind that certain conditions must be met to perform control examinations. Loss of too many hair shafts may lead to difficulties in matching the baseline follicular map; therefore, the length of the follow-up period should correlate with hair loss activity.

A number of limitations of this study have been noted. First, the number of patients was small. Second, the time interval of 12 months between the initial and follow-up examination may be considered short. Third, only one scalp spot was analyzed in each patient. Lastly, as only a small group of FFA patients has been observed, a variety of other patterns might appear in the future as more patients are studied.

The objective of this study was to propose means for measurement of FFA progression. Our study presents a method of analysis of this phenomenon for a chosen spot on the scalp, and obtained results should not be considered representative for all affected scalp areas. Of course, examination of several spots would be more informative on overall FFA progression. Our procedure may be considered too cumbersome for daily clinical practice but provides exact, reproducible, and detailed information on actual hair loss which may be extremely helpful in clinical studies.

## Conclusion

In conclusion, trichoscopy with H2H matching allows the detection and measurement of hair loss also in FFA patients where no disease progression is clinically observed. The hair density profile may be a useful tool to characterize different disease progression patterns. The HRE is potentially a very accurate and sensitive parameter to quantify local hair loss progression in FFA.

## Statement of Ethics

Ethical approval was not required for this study in accordance with national guidelines as trichoscopy is a standard procedure in hair disease monitoring. All subjects have given their written informed consent to participate in the study.

## Conflict of Interest Statement

Michał Kasprzak is a founder and CEO of TrichoLAB. Justyna Sicińska is a consultant for TrichoLAB and FotoFinder and a family member of TrichoLAB founders. Irena Walecka declares no conflict of interest.

## Funding Sources

The authors declared that no grants were involved in supporting this work. The analysis of trichoscopic images by TrichoLAB was performed free of charge.

## Author Contributions

Justyna Sicińska designed the study, acquired the data, prepared the draft, wrote and revised the article, and approved the final version to be published. Michał Kasprzak acquired the data, prepared the draft, analyzed and interpreted data for the work, and approved the final version to be published. Irena Walecka designed the study, prepared the draft, revised the draft, interpreted data for the work, and approved the final version to be published.

## Data Availability Statement

Underlying data. Open Science Framework. HRE. https://doi.org/10.17605/OSF.IO/2RYDA [27]. The project contains the following underlying data: data are available under the terms of the Creative Commons Attribution 4.0 International license (CC-BY 4.0).
